# Global climate shift in 1970s causes a significant worldwide increase in precipitation extremes

**DOI:** 10.1038/s41598-021-90854-8

**Published:** 2021-06-02

**Authors:** Subharthi Sarkar, Rajib Maity

**Affiliations:** Department of Civil Engineering, Indian Institute of Technology Kharagpur, Kharagpur, West Bengal 721302 India

**Keywords:** Hydrology, Climate change, Hydrology

## Abstract

The shift in climate regimes around 1970s caused an overall enhancement of precipitation extremes across the globe with a specific spatial distribution pattern. We used gridded observational-reanalysis precipitation dataset and two important extreme precipitation measures, namely Annual Maximum Daily Precipitation (AMDP) and Probable Maximum Precipitation (PMP). AMDP is reported to increase for almost two-third of the global land area. The variability of AMDP is found to increase more than its mean that eventually results in increased PMP almost worldwide, less near equator and maximum around mid-latitudes. Continent-wise, such increase in AMDP and PMP is true for all continents except some parts of Africa. The zone-wise analysis (dividing the globe into nine precipitation zones) reveals that zones of ‘moderate precipitation’ and ‘moderate seasonality’ exhibit the maximum increases in PMP. Recent increased in pole-ward heat and moisture transport as a result of Arctic Amplification may be associated with such spatial redistribution of precipitation extremes in the northern hemisphere.

## Introduction

Global climate change and consequent warming of atmospheric system has altered the regular spatio-temporal distribution of precipitation^1–3^, especially the regime of extreme precipitation all over the world^4^. To be specific, researchers have detected some abrupt, substantial, and persistent changes or ‘shift’ in the state of natural climatic systems around the 1970s in different parts of the globe^5–14^. Generally, this phenomenon is referred as ‘global shift in climatic regime’ in literature, which manifests itself as sudden jump in several hydroclimatic variables such as, temperature, air pressure, wind field, precipitation etc.^15^. However, its impact on spatial distribution of precipitation extremes is not well-explored, in spite of its potential socio-economic implications. Towards this, we have considered two very important measures of precipitation extremes from hydrological design point of view, namely Annual Maximum Daily Precipitation (AMDP) and Probable Maximum Precipitation (PMP). AMDP, the maximum one day precipitation over a year is extensively used for several hydrological analysis such as flood risk assessments^16–18^, hydrological response of streams in a river basin, soil erosion, dams silting etc.^19–21^, which all are likely to alter under this changing scenario of climate. On the other hand, usefulness of PMP, which designates the physical upper limit of precipitation at a particular place, is oblivious to state. It ranges from estimation of Probable Maximum Flood (PMF)^22^ to the design of spillway of large dams^23^ to the design of major energy infrastructures like nuclear power plants^24^, and so on. All these water-energy infrastructures are generally associated with high risk, as they may cause devastating repercussions upon their failure. Hence, a rational concept like PMP is commonly used as the design criterion to ensure a negligible probability of exceedance^25^. All these aforementioned high-risk, high-cost infrastructures possess a very long life span (more than 100 to 500 years), ensuring their experience of future changes in climate in their lifespan. Consequently, the design risk and reliability of those infrastructures are also expected to alter over the years^26–28^. Hence, in order to ensure serviceability and longevity of those structures in future, proper systematic analysis on temporal evolution of precipitation extremes such as, AMDP and PMP on global, as well as regional scale have become indispensable in the context of climate change.

In brief, this study aims to explore the potential impacts of the shift in global climate regime in 1970s on the overall nature of precipitation extremes. Towards this, a global and continental scale assessment of AMDP is conducted to capture the likely spatio-temporal changes w.r.t. its different statistical properties such as, mean and variability. Further, the entire global land area is divided into nine zones of uniform precipitation characteristics to interpret the results at zone level. The year 1978 is considered as the transition year to identify the changes due to global shift in climate regime in the post-1978 period (1978–2012) relative to the pre-1978 period (1948–1977). Moreover, the global PMP maps are prepared for pre- and post-1978 period to capture the spatio-temporal changes in PMP. The relative changes in PMP is also properly interpreted at global, as well as continental and zone level. These PMP maps, especially the recent one (post-1978) will provide an important information for the design engineers and hydro-meteorologists for revised planning and designing various major water-energy infrastructures in the context of climate change.

## Results

### Zoning based on precipitation characteristics

The zone-wise analysis is done by dividing the entire global land area into nine distinct precipitation zones, following a recent work^29–31^. This classification is done based on the average annual precipitation (*P*) and seasonal variation of monthly precipitation over the base period, i.e. pre-1978 period (1948–1977). To quantify the seasonality, an information theory based metric, named Apportionment Entropy (AE) is utilised, which provides a descriptive non-parametric measure of the seasonal variation for any data, and higher the magnitude of AE, less seasonal the precipitation data is, and vice versa. (see “Methods” section for further details).

**Figure 1:**
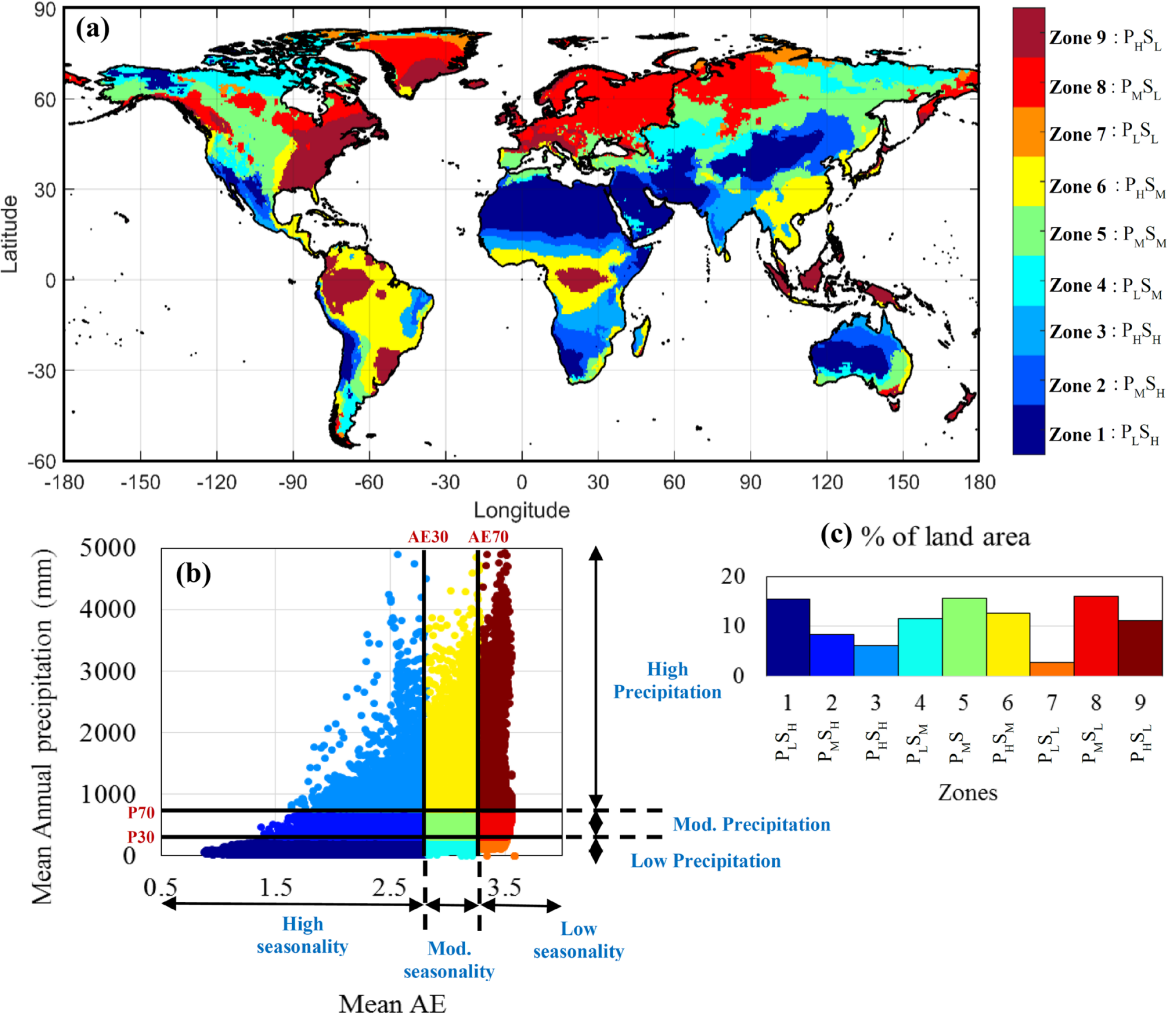
Zoning based on precipitation characteristics. (**a**) Nine precipitation zones based on annual precipitation and seasonality used in this study, (**b**) scatter plot of mean annual precipitation and mean AE, and corresponding delineation of nine zones, (**c**) Percentage of land area occupied by each precipitation zones. The figure was produced using MATLAB software (version R2021a, URL: https://in.mathworks.com).

The spatial distribution of these nine precipitation zones is shown in Fig. 1a. The classification is done based on the scatter plot between *P* and AE, which is delineated into nine different zones (shown by different colours), by defining two separate thresholds (30th and 70th percentile, i.e., *P*_*30*_*, P*_*70*_ and *AE*_*30*_*, AE*_*70*_) along both the axes, as shown in Fig. 1b. Thus in this classification approach, the characteristics of both annual precipitation magnitude and seasonal variability are coupled into nine distinct precipitation zones. The percentage of global land area occupied by each zone is shown in Fig. 1c. The detailed insight of this classification is shown in Table 1, including the criterion for classification, zone number, zone full name, and corresponding abbreviations. For example, for a grid point, if the average annual precipitation (*P*) is higher than *P*_*70*_ and average AE value lies between AE_30_ and AE_70_, then that particular grid point falls in ‘zone 6’, i.e. the High Precipitation-Moderate Seasonality zone (P_H_S_M_).

**Table1:** Details of the zoning based on mean annual precipitation (*P*) and AE over the pre-1978 period, used in this study.

Zone number	Criteria for classification	Zone name	Abbreviation
Zone-1	P < P_30_ and AE < AE_30_	Low Precipitation High Seasonality	P_L_S_H_
Zone-2	P_30_ < P < P_70_ and AE < AE_30_	Mod. Precipitation High Seasonality	P_M_S_H_
Zone-3	P > P_70_ and AE < AE_30_	High Precipitation High Seasonality	P_H_S_H_
Zone-4	P < P_30_ and AE_30_ < AE < AE_70_	Low Precipitation Mod. Seasonality	P_L_S_M_
Zone-5	P_30_ < P < P_70_ and AE_30_ < AE < AE_70_	Mid Precipitation Mod. Seasonality	P_M_S_M_
Zone-6	P > P_70_ and AE_30_ < AE < AE_70_	High Precipitation Mod. Seasonality	P_H_S_M_
Zone-7	P < P_30_ and AE > AE_70_	Low Precipitation Low Seasonality	P_L_S_L_
Zone-8	P_30_ < P < P_70_ and AE > AE_70_	Mod. Precipitation Low Seasonality	P_M_S_L_
Zone-9	P > P_70_ and AE > AE_70_	High Precipitation Low Seasonality	P_H_S_L_

Further, all these nine zones can be categorised into some broader precipitation regimes for ease in discussing and interpreting the results. For example, Zone 1, zone 2 and zone 3 can be clubbed into a single category named the ‘High seasonality zone’. Likewise, zone 4, 5 and 6 make the ‘Moderate seasonality zone’, and zone 7, 8 and 9 constitute the ‘Low seasonality zone’. On the other hand, zones 3, 6 and 9 make the ‘High precipitation zone’, zones 2, 5 and 8 make the ‘Moderate precipitation zone’, and finally zone 1, 4 and 7 constitute the ‘Low precipitation zone’.

From Fig. 1a, we observe that, ‘High seasonality zone’ can be mostly found in the monsoon-dominant regions such as, Indian subcontinent, Middle East, some parts of central Asia, Northern Australia, and much of north-central and south-central Africa. On the other hand, the ‘Low seasonality zone’ can be found in almost entire Europe, Western North America, some parts of Northern South America, central Africa, Indonesia etc. Likewise, ‘Moderate precipitation zone’ can be observed in most of central North America, Europe, and scattered places in southern parts of South America, northern and central Asia, southern Africa and southern Australia. Similarly, remaining zones can also be identified from the Fig. 1a.

### Analysis of AMDP

**Figure 2:**
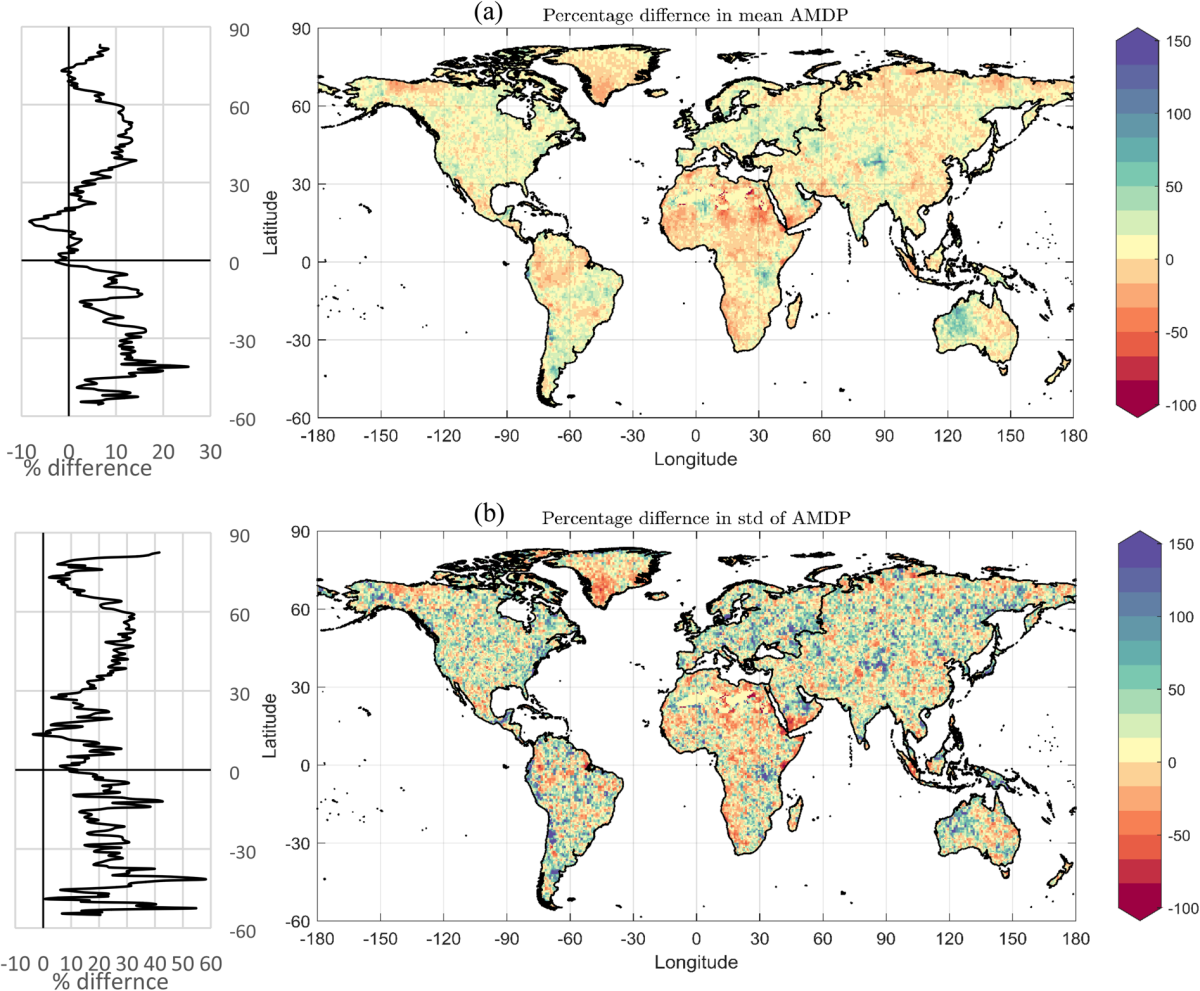
Global Spatial Distribution of changes in AMDP and its distribution along latitude. (**a**) Percentage difference in mean AMDP and (**b**) Percentage difference in standard deviation of AMDP, in post-1978 (1978–2012) period, w.r.t. pre-1978 (1948–1977) period, along with the average percentage changes along the latitude (left panel). The figure was produced using MATLAB software (version R2021a, URL: https://in.mathworks.com).

**Figure 3:**
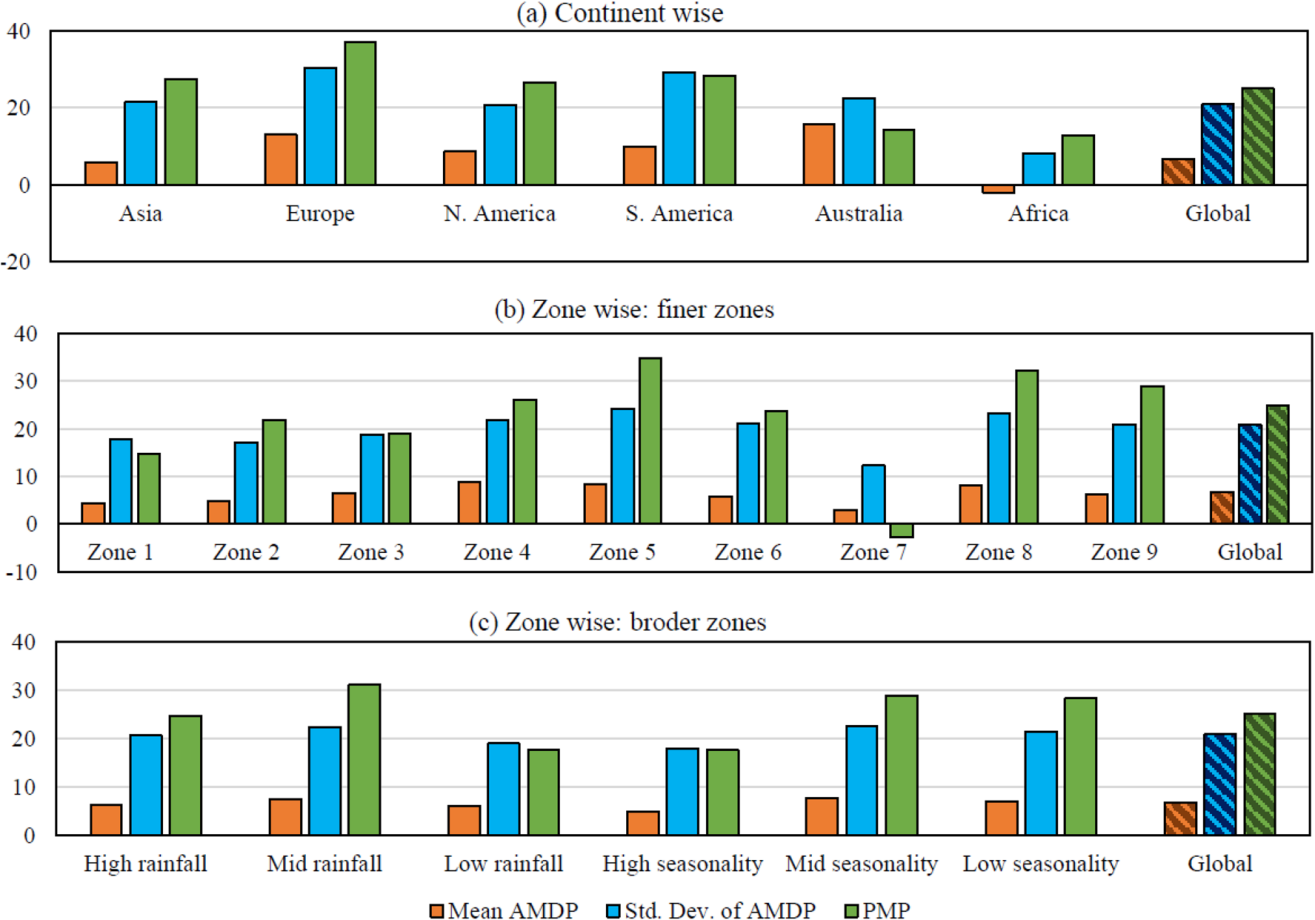
Average percentage change in mean and standard deviation of AMDP and PMP in the post-1978 period with respect to pre-1978 period at global, continental and zone level. The hatched bars indicate the average percentage changes at global scale for each subplot.

We investigate on changes in two key statistical features of AMDP, i.e., mean and standard deviation. The changes in the post-1978 period relative to pre-1978 period is expressed in terms of percentage changes, and the corresponding global spatial map of changes in both mean and standard deviation of AMDP is shown in Fig. 2a,b, respectively. The blue (various shades) portions in this figure indicate the places with observed increases, and the red (various shades) patches indicate decrease in the post-1978 period. The quantitative outcomes of this comparative study at global and continental scale are shown in Supplementary Table 1, and presented here as bar diagram in Fig. 3. The results reveal that the mean AMDP has increased by 6.73% on an average all over the globe, and it is true for more than two-third (67.28%) of the global land area. On the continental scale, Europe, Australia and two Americas exhibit considerable amount of increase in mean AMDP in post-1978 period, both in terms of average increase and percentage of land-area showing the increase. Moreover, all these four above-mentioned continents show increase in mean AMDP, higher than the global average value (i.e., 6.73%). In case of the remaining two continents, viz. Asia and Africa, the increase in mean AMDP is found to be lower than the global average value. Even, Africa shows an overall reduction in mean AMDP values by 2.01%, and such reduction is observed for more than 50% of its land area. All these continental scale observations can be visually confirmed from the Fig. 2a. Further, for the global spatial distribution map of mean and standard deviation of AMDP, readers may see the Supplementary Figs. 1 and 2, respectively.

Likewise, in case of standard deviation of AMDP, we found it to be increased by 20.84% at global scale post the year 1978, which is almost three times higher than the average global increase in mean AMDP (i.e., 6.73%). However, in terms of percentage of land area showing such increase, it (66.13% of global land area) is lower compared to that of mean AMDP (67.28% of global land area). In addition, comparing Fig. 2a,b we see that, in most of the cases, the places with increase in mean AMDP are showing increase in standard deviation, as well. However, it is not always true. From the continental scale analysis, we found that, Europe, South America, Asia and Australia are showing significant increase in standard deviation of AMDP, which is higher than the global average value. Though unlike mean AMDP, Africa is not showing overall decreasing trend in standard deviation of AMDP, the extent of increase (8.20%) is much below the global average value. Furthermore from the latitudinal distribution of the percentage changes in mean and standard deviation of AMDP, we found that, the extent of increase is comparatively lower around the tropics (sometimes even decrease is observed), and reaches the peak mostly around mid-latitudes.

This global and continental scale comparative study of AMDP is followed by a zone-wise analysis to see how this overall increase in AMDP got reflected in different zones of precipitation. The results are shown in Supplementary Table 2, and here as bar diagram in Fig. 3b,c. From the results, we can see that the zone-4 (P_L_S_M_), zone-5 (P_M_S_M_), and zone-8 (P_M_S_L_) are showing maximum amount of increase in mean AMDP. On the other hand, zone-1 (P_L_S_H_), zone-2 (P_M_S_H_), and zone-7 (P_L_S_L_) exhibit the least amount of increase. Considering the broader precipitation zones (Fig. 3c), we found that, the maximum amount of increase in mean AMDP is observed in case of both Moderate zones, viz., Moderate precipitation zone and Moderate seasonality zone. The High precipitation zones and low seasonality zones also exhibit good amount of increase. However, the low precipitation zones and high seasonality zones show the least amount of increase among all other zones.

Similar observations hold true for standard deviation of AMDP as well. Additionally, it can be noticed that for all zones, the magnitude of increase in standard deviation of AMDP is substantially higher (two–three fold), as compared to the increase in mean AMDP. This signifies that, not only the mean AMDP, its variability has also increased significantly for each zone of precipitation due to the global shift in climate regimes in 1970s.

**Figure 4:**
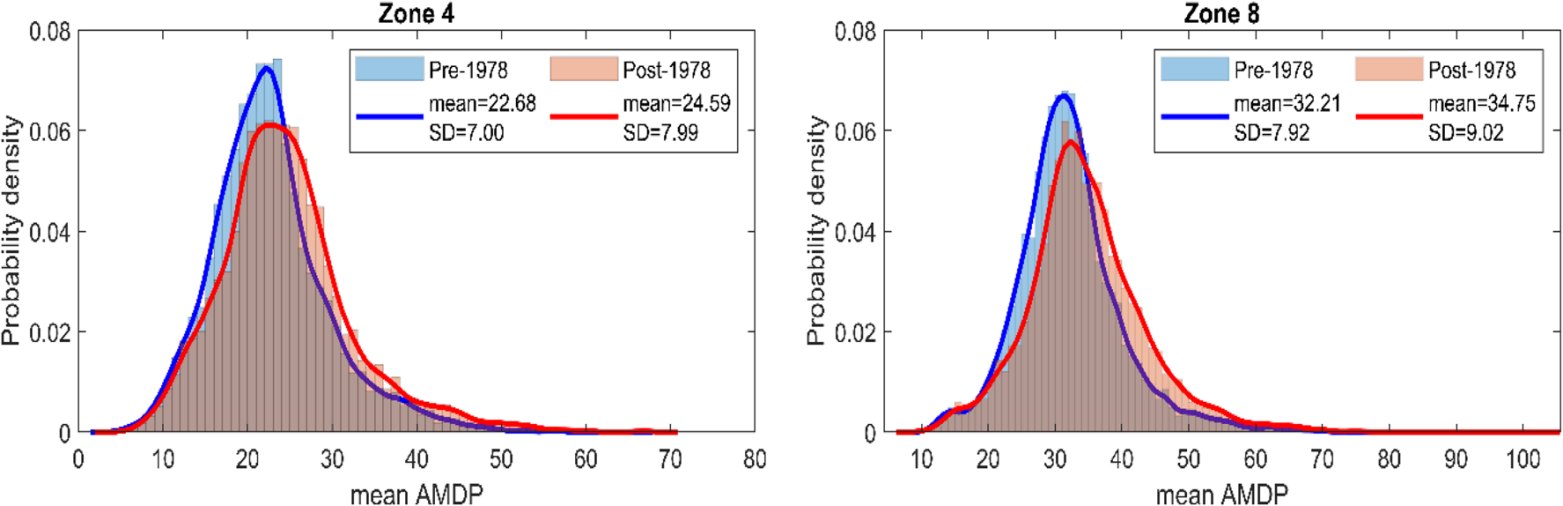
Zone-wise histogram for mean AMDP and fitted normal Kernal distribution function for Pre- and post-1978 period for two representative precipitation zones, zone-4 (P_M_S_L_) and zone-8 (P_L_S_M_). The spatial avearge (in mm) and standard deviation (in mm) of the mean AMDP values for both zones are provided in the inset of each figure.

To further contribute to the aforementioned results, histogram is plotted with the mean AMDP ($\overline{X}_{N}$) values for all grid-points within a zone, followed by fitting a normal Kernel density function to obtain the shape of the underlying frequency distribution. Then the corresponding spatial average and standard deviation values are compared between their pre- and post-1978 values, and shown in Supplementary Table 3 for all nine zones. For visual understanding, the histogram and corresponding fitted distribution are shown here in Fig. 4 for two representative precipitation zones, i.e., zone-4 (P_M_S_L_) and zone-8 (P_L_S_M_) for brevity. We can observe how the probability density function is being shifted toward the higher mean AMDP values, and being flattened as well in the post-1978 period. Additionally, many new records of mean AMDP values are observed in these zones after 1978, eventually resulting in increase in spatial average and standard deviation simultaneously. Similar observations can be confirmed for all other remaining zones from the Supplementary Table 3.

### Analysis of PMP

Among several methods available in literature for estimation of PMP^32–36^, Hershfield statistical method^37,38^ is considered as a convenient, popular and efficient tool for quick estimation of PMP, and hence used in this study. The most crucial step in Hershfield method is the precise estimation of frequency factor (*K*), for which one exponential upper envelope curve was suggested^38^. However, a recent study^39^ argued that, such consideration of single upper envelope curve may lead to overestimation of PMP, especially for the regions with climatologically low extreme precipitation, and proposed a composite upper envelope curve instead (see “Methods” section for further details). Here we apply the upgraded technique of enveloping for estimating PMP as per Hershfield method.

**Figure 5:**
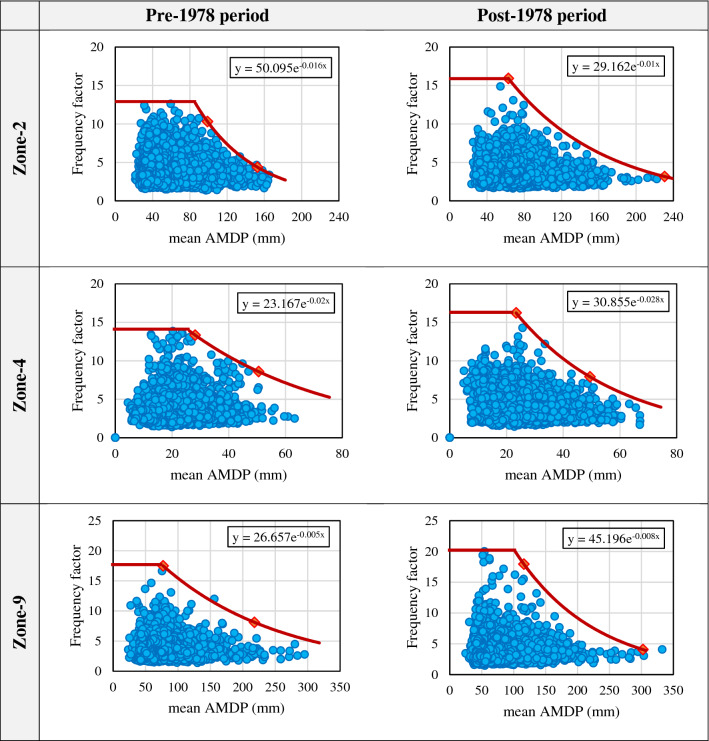
The scatter plot and upper envelope curve for three representative zones, viz. zone-2, zone-4, and zone-9, for pre- and post-1978 period. The equation of the exponential portion of the envelope curves are given in the inset of each subplot.

Rather than a single global envelope curve, individual envelope curves are developed for each of those nine precipitation zones to avoid further overestimation. However, for brevity, the envelope curves are shown only for three precipitation zones in Fig. 5 (red curve), for pre- and post-1978 period. These composite envelope curves clearly have two different parts; one straight-line portion, parallel to x-axis with the maximum value of *K* (i.e., *K*_*m*_), and one exponentially decaying curve. To develop this exponential part, some points near the upper part of the cluster (shown as orange diamond points in Fig. 5) from the scatter plot are chosen, and an exponential curve is fitted, whose equation is provided in the inset. Using these envelope curves, frequency factor at each grid point is re-estimated and used for estimation of PMP as per Hershfield method (see “Methods” section for details).

**Figure 6:**
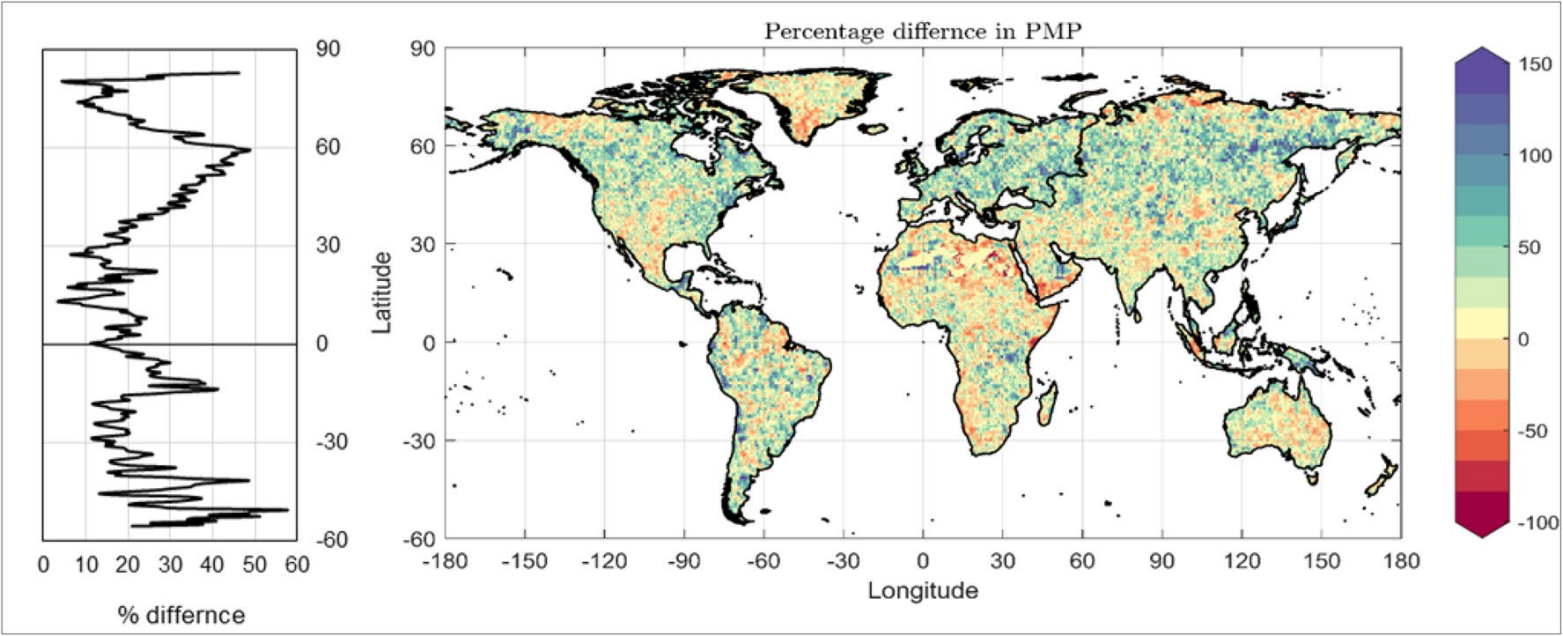
Global Spatial Distribution of changes in PMP and its distribution along latitude. Percentage difference in PMP, in post-1978 (1978–2012) period, w.r.t. pre-1978 (1948–1977) period (right), and the average percentage changes along the latitude (left). The figure was produced using MATLAB software (version R2021a, URL: https://in.mathworks.com).

Following this method, global PMP maps are developed for pre- and post-1978 period, and shown in Supplementary Fig. 3. The percentage difference between the pre-and post-1978 PMP estimates are shown as global spatial distribution in Fig. 6. In this figure, different shades of blue portions indicate the places where increase in PMP are observed, and different shades of red portions indicate decrease in PMP after the year 1978. The quantitative results of this comparative analysis is provided in Supplementary Tables 4 and 5 at global, continental, and zone level, and shown here as bar plot in Fig. 3. The analysis reveals that the global PMP has increased by 25.04% on an average all over the world, and such increase is observed for more than three-fourth of the global land area (i.e. 76.44%). Similar to AMDP, here also higher extent of increase in PMP is observed in the mid-latitudes compared to tropics.

Coming to the continental scale results (Fig. 3a), Europe shows the maximum amount of increase in PMP, i.e. 36.99%, which is true for almost its entire land area (88.47%). Such a high level of increase in PMP can be explained by revisiting the results of comparative study on mean and standard deviation of AMDP. There also, a substantial increase in mean and especially in standard deviation of AMDP was observed for Europe, which eventually resulted in this level of increase in PMP in the post-1978 period. In terms of extent of increase in PMP, Europe is subsequently followed by South America (28.33%), Asia (27.48%) and North America (26.47%). All these three continents show increase in PMP, higher than the global average value, and more than 75% of their land area exhibits such increase. In case of Australia (14.23%), and Africa (12.82%), the extent of increase is considerably lower than the global average value. The results of Africa can again be explained by using the results of comparative study on mean and standard deviation of AMDP, where Africa showed very small amount of increase in standard deviation, and some decrease in mean AMDP. However, though Australia showed a good amount of increase in both mean and standard deviation of AMDP, the increase in PMP is not at the similar level. Probably because, majority of Australia falls in the High seasonality region, where the extent of increase is least, such observations are reported. Nonetheless, an overall increase in PMP is reported in each continent in the post-1978 period, which can be considered as an impact of global shift in climate regime in the 1970s.

In case of zone-wise analysis of PMP (Fig. 3b,c), similar to the results of mean and standard deviation of AMDP, we see that the zone-4 (P_L_S_M_), zone-5 (P_M_S_M_), zone-8 (P_M_S_L_) and zone-9 (P_H_S_L_) are showing maximum amount of increase in PMP over approximately 80% or more area. On the other hand, zone-1 (P_L_S_H_), zone-2 (P_M_S_H_), and zone-3 (P_H_S_H_) exhibit comparatively smaller amount of increase. Meanwhile, zone-7 (P_L_S_L_) shows some reduction in PMP (-2.83%), which may be attributed from a very low-level increase in mean and standard deviation of AMDP in zone-7. Considering the broader precipitation zones, we found that, the maximum amount of increase in PMP is observed in case of both the Moderate zones; viz., Moderate precipitation zone (31.13%) and Moderate seasonality zone (28.8%). The extents of increase in PMP in Low seasonality zones (28.25%) is marginally lesser than that of Moderate seasonality zone, but in case of High seasonality zone (17.6%), it is considerably lower. Similarly, the High precipitation zones (24.77%) and Low precipitation zones (17.71%) exhibit lesser extent of increase in PMP compared to Moderate precipitation zones. Keeping the exception of zone-7 aside, the overall global increase in PMP is also well captured by all the precipitation zones, but of different spatio-temporal extents.

## Discussion

An overall enhancement in both AMDP and PMP is detected in the post-1978 period at global, continental and zone level, which can be considered as a significant impact of global shift in climate regime in 1970s. All the continents (except Africa) exhibit increase in mean AMDP, but of different spatio-temporal extents. Additionally, we also notice that the average increase in standard deviation of AMDP is substantially higher (two to three times, in some cases) than that in mean AMDP for all continents, and eventually for the entire globe. This provides an important insight to the changing nature of precipitation extremes like AMDP, i.e., not only the mean AMDP has increased, but its variability has also increased significantly under the impact of global shift in climate regimes in 1970s. Approximately two-third of the global land area experiences such increase.

Owing to such increase in mean and standard deviation of AMDP, PMP has increased all over the globe in the post-1978 period. Increase in standard deviation of AMDP implies increased variability of daily extreme precipitation on annual scale. Increased variability (or ‘dispersion’ in statistical terms) increases the range of the AMDP series, which in turn leads to increase in the upper limit of precipitation, and eventually the PMP increases. This physical justification can be mathematically confirmed from Eq. (4). This equation for estimation of PMP can be considered as a multiple linear regression equation, with the dependent variable PMP, two independent variables- mean ($\overline{X}_{N}$) and standard deviation (*S*_*N*_) of AMDP, and zero intercept. The regression coefficient for $\overline{X}_{N}$ is one, whereas the same for *S*_*N*_ is frequency factor (*K*), which is always more than one (see Fig. 5). Therefore, even if percentage increase for mean and standard deviation are exactly same, the change in standard deviation will have more profound impact on PMP because of the factor *K*. In other words, it can be said that, PMP is more sensitive towards change in standard deviation of AMDP than the mean AMDP. Moreover, we found that the increase in standard deviation of PMP is approximately two–three times higher than that of mean AMDP; hence, its impact on PMP will be even more pronounced, which have been reported at global, continental and zone level in this study.

On the continental scale, Europe shows the maximum amount of increase in PMP in terms of both the percentage increase and the percentage of area showing the increase. Apart Europe, Asia, two Americas show good amount of increase in PMP after the year 1978. As expected, the extent of increase is least in case of Africa, among all the continents. Overall, more than three-fourth of the global land area shows increase in PMP, with an average of approx. 25% increase. Interestingly, the extent of increase is found to be maximum around the mid-latitudes. Such enhancement of precipitation extremes in mid-latitudes may be associated with the Artic Amplification (AA), i.e., warming of Arctic Circle at almost twice the global average rate, following some previous studies^40–45^. The rapid Arctic warming has contributed to faster melting of Arctic sea ice and spring snow cover, eventually resulting in reduced meridional temperature gradients connected to atmospheric circulation, and thus affecting the mid-latitude weather and climate significantly^40^. However, it requires further study to draw any such conclusion firmly.

The global increasing pattern of AMDP and PMP is well reflected in different precipitation zones across the globe, especially in case of Moderate precipitation and Moderate seasonality zones. Slightly lesser increase is captured in the High precipitation and Low seasonality zones. On the other hand, the Low precipitation zones and the monsoon-dominant High seasonality zones show the least amount of increase. Thus in general, the places which are climatologically wet (high precipitation) and having consistent supply of water (low seasonality) throughout the year are more susceptible to changes in extreme precipitation, than the climatologically dry places (low precipitation) with seasonal availability of water (high seasonality).

To conclude, the characteristics of precipitation extremes like AMDP and PMP have altered significantly all over the world as a possible impact of global shift in climate regime in 1970s. The concomitant increases in mean and standard deviation of AMDP has resulted in an overall increase in PMP across the globe. Such temporal changes, especially in PMP must be considered in the revised planning, designing and future risk assessment of any major high-risk water resources infrastructure under climate change adaptation and mitigation strategies.

### Data

A long-term (1948–2012), high-resolution, gridded (0.5° latitude $\times$ 0.5° longitude), global daily precipitation dataset, provided by ‘Terrestrial Hydrology Research Group-Princeton University’ is used in this study. This consistent and high-quality dataset was developed^46^ by blending the latest available global observed meteorological datasets with the state-of-the-art National Centres for Environmental Prediction–National Centre for Atmospheric Research (NCEP–NCAR) reanalysis data. This was followed by a set of validation against some independent observational data sources to quantify the bias (i.e. the residual error), and de-bias it accordingly. Thus, this approach retains the consistency and continuity of the reanalysis product, but constrains it to the best available observation datasets. Hence, we have used this gridded dataset in our analysis as a proxy to the actual station-observed data. Moreover, use of gridded data helps to develop the final gridded global PMP maps, and interpret the corresponding results in an easier and handy way than the observational data, which are generally available at coarser resolutions and reduced spatial and temporal extents. For further detailed information on this gridded data and its detailed development procedure, readers can refer to the original publication^46^.

To capture the impact of global shift in climate regime, we split this 65 years’ data (1948–2012) into two parts, viz. Pre-1978 (1948–1977) and Post-1978 (1978–2012), considering the year 1978 as the year of transition. Many studies indicated late 1970s as the time for some abrupt shift in global climate regime. Global averaged surface temperature of Earth (see Supplementary Fig. 4) since 1880 indicates a clear increasing trend of temperature from late 1970s. Moreover, a standard window of 30 year is recommended by World Meteorological Organisation (WMO) to capture any climatological changes. Therefore, to ensure the length of base-period (1948–1977) as 30 year, 1978 becomes an obvious choice. Thus, the year 1978 is chosen as the transition year to capture the potential impacts of global shift in climate regime on precipitation extremes like AMDP and PMP.

## Methods

### Zoning based on precipitation characteristics

To perform the zone-wise analysis of AMDP and PMP, the entire global land area is divided into nine distinct precipitation zones in this study, following a recent work^29^. This classification is done based on the average annual precipitation (*P*) and seasonal variation of monthly precipitation over the base period, i.e. pre-1978 period (1948–1977). To quantify the seasonality, an information theory based metric, named Apportionment Entropy (AE) is utilised, which provides a descriptive non-parametric measure of the seasonal variation for any data (here, monthly precipitation). The value of AE for *k*th year is calculated by

1 $$AE_{k} = \sum\limits_{i = 1}^{12} {\left( {p_{ik} /P_{k} } \right)\log_{2} \left( {p_{ik} /P_{k} } \right)}$$

where *p*_*ik*_ is the monthly precipitation for *i*th month in *k*th year and *P*_*k*_ is the total annual precipitation for the *k*th year, hence given by

2 $$P_{k} = \sum\limits_{i = 1}^{12} {p_{ik} }$$

Theoretically, the magnitude of AE may range between zero and the maximum value of $\log_{2} 12\left( { \approx 3.585} \right)$. Higher the magnitude of AE, less seasonal the precipitation data is, and vice versa. Next, the average values of annual precipitation and AE are calculated for all grid points over the 30-year long base period, and plotted as a scatter plot, as shown in Fig. 1b. This scatter plot is then delineated into nine different zones (shown by different colours), using two intersecting dividing lines that pass through two separate thresholds of both the variables (30th and 70th percentile, i.e., P_30_, P_70_ and AE_30_, AE_70_). The 30th and 70th percentiles are chosen based upon the wet and dry region definitions available in various earlier literatures^47–50^. Thus in this classification approach, the characteristics of both annual precipitation magnitude and seasonal variability are coupled into nine distinct precipitation zones.

### Analysis of AMDP and comparative study

From the time-series of daily precipitation records, the annual maximum values are filtered out for all the years, and the AMDP series are prepared at each grid point. If *x*_*ik*_ is the magnitude of precipitation on *i*th day in *k*th year, then AMDP for the *k*th year is expressed as,

3 $$X_{k} = \max (x_{ik} )\;{\text{for}}\;k = 1,2,3, \ldots ,N$$

where *N* is the total number of years considered. After extracting the AMDP series for both pre- and post-1978 period, the mean AMDP $\left( {\overline{X}_{N} = \frac{1}{N}\sum\limits_{j = 1}^{N} {X_{j} } } \right)$ is calculated at each grid-point over the world, followed by a comparative study between the pre- and post-1978 mean values. This comparative study is done by calculating the change in mean AMDP values in post-1978 period, relative to pre-1978 period, and expressed in terms of percentage change. A similar comparative analysis is carried out for the standard deviation of AMDP $\left( {S_{N} = \sqrt {\frac{1}{N - 1}\sum\limits_{j = 1}^{N} {(X_{j} - \overline{X}_{N} )} } } \right)$ series to check how the variability of such extremes got affected due to global climatic shift, along with the mean. This comparative study will identify whether the AMDP values are getting more dispersed with time or not, or in other words, whether the variability in AMDP series is increasing with time or not, and if yes, then at what extent. Overall, this comparative study of mean AMDP along with its standard deviation will provide a holistic idea on possible changes in the characteristics of precipitation extremes (here, AMDP) due to global shift in climate regime on the global scale.

### Estimation of PMP and comparative analysis

Here we used Hershfield method (1965) for estimation of PMP, with an upgraded technique for enveloping by a recent study by Sarkar and Maity (2020)^39^. According to this method, PMP at any location can be estimated using the general frequency equation as suggested by Chow^51^,

4 $$X_{PMP} = \overline{X}_{N} + K \times S_{N}$$

where *X*_*PMP*_ is the PMP estimate of that location, $\overline{X}_{N}$ is the mean AMDP over *N* years at that location, *S*_*N*_ is the standard deviation of the AMDP series at that location, *K* is the frequency factor for estimating PMP, which can be determined from the following equation, proposed by Hershfield (1961),

5 $$K = \frac{{X_{m} - \overline{X}_{N - 1} }}{{S_{N - 1} }}$$

where *X*_*m*_ is the maximum value in the AMDP series at that location $\overline{X}_{N - 1}$ and $S_{N - 1}$ are the mean and standard deviation of the AMDP series, respectively for (*N*-1) years after removing the year with the maximum value, i.e.*X*_*m*_.

Next, a composite upper envelope curve for $K$ is prepared following Sarkar and Maity (2020). According to this method, firstly the scatter plot between $K$ and $\overline{X}_{N}$ is prepared for the zone of interest. A typical example of such scatter plot is shown in Supplementary Fig. 5, modified and reproduced from Sarkar and Maity (2020). The composite upper envelope curve shown on this scatter plot (the red curve) has two segments; one straight-line part parallel to X-axis (i.e. the axis showing mean AMDP), and one exponentially decaying portion. The straight-line portion and the exponential curve intersects at mean AMDP ($\overline{X}_{N}$) = $\overline{X}_{N}^{t}$ (t refers to transition point) and frequency factor (*K*) = *K*_*m*_, where $\overline{X}_{N}^{t}$ denotes the mean AMDP at transition point, and *K*_*m*_ is the maximum value of *K* within the study area. Thus, the equation of this composite envelope curve is given by:

6 $$K = \left\{ {\begin{array}{*{20}l} {K_{m} } \hfill & {0 < \overline{X}_{N} < \overline{X}_{N}^{t} } \hfill \\ {K_{m} e -^{{d\left( {\overline{X}_{N} - \overline{X}_{N}^{t} } \right)}} } \hfill & {\overline{X}_{N} > \overline{X}_{N}^{t} } \hfill \\ \end{array} } \right.$$

In the above equation, a factor ‘*d*’ is there, which determines the slope of the exponentially decreasing portion, and is a function of the duration of interest and the study area. Then, the new value of frequency factor can be read from this envelope curve corresponding to the $\overline{X}_{N}$ for a particular grid point, and then PMP can be estimated at that grid point using Eq. (4). Finally, a comparative analysis is carried out between the pre- and post-1978 PMP estimates to capture change.

## Supplementary information

Supplementary Information.

## Data Availability

High-resolution, gridded (0.5° latitude $\times$ 0.5° longitude), global daily precipitation dataset, is procured from ‘Terrestrial Hydrology Research Group-Princeton University’ (URL: https://hydrology.princeton.edu/data.lsm.php).

## Code availability

The MATLAB codes developed for the analysis and these are available for research/academic purposes on request from the authors.
