# Supplementary material for: Global climate shift in 1970s causes a significant worldwide increase in precipitation extremes
Source: Sci Rep. 2021 Jun 2;11:11574. doi: 10.1038/s41598-021-90854-8 (PMC8172922; doi:10.1038/s41598-021-90854-8)
Supplement: Supplementary file 1 — Supplementary Information. [file 41598_2021_90854_MOESM1_ESM.docx]

Supplementary Information

**Global climate shift in 1970s causes a significant worldwide increase in precipitation extremes**

Subharthi Sarkar and Rajib Maity*

Department of Civil Engineering, Indian Institute of Technology Kharagpur

Kharagpur – 721302, West Bengal, India

*Corresponding Author: email – [rajib@civil.iitkgp.ac.in](mailto:rajib@civil.iitkgp.ac.in); [rajibmaity@gmail.com](mailto:rajibmaity@gmail.com)

This document contents all the supplementary tables and figures mentioned in the main article.

**Supplementary Table 1**: The Global and continental scale increase in mean and standard deviation of AMDP and the percentage of area showing the increase.

| **Continents** | **Mean AMDP** | | **Std. Dev. of AMDP** | |
| --- | --- | --- | --- | --- |
|  | **Avg. % increase** | **% area** | **Avg. % increase** | **% area** |
| **Global** | 6.73 | 67.28 | 20.84 | 66.13 |
| **Asia** | 5.82 | 65.81 | 21.49 | 67.26 |
| **Europe** | 13.06 | 84.52 | 30.33 | 75.31 |
| **N. America** | 8.59 | 75.22 | 20.75 | 67.30 |
| **S. America** | 10.01 | 70.61 | 29.22 | 69.78 |
| **Australia** | 15.83 | 78.01 | 22.45 | 67.45 |
| **Africa** | -2.01 | 43.11 | 8.20 | 53.93 |

**Supplementary Table 2**: Zone-wise increase in mean and standard deviation of AMDP and the percentage of area showing the increase.

| **Precipitation Zones** | **Mean AMDP** | | **Std. Dev. of AMDP** | |
| --- | --- | --- | --- | --- |
|  | **Avg. % increase** | **% area** | **Avg. % increase** | **% area** |
| **Zone 1 (P_L_S_H_)** | 4.46 | 57.31 | 17.96 | 60.76 |
| **Zone 2 (P_M_S_H_)** | 4.95 | 61.76 | 17.13 | 64.35 |
| **Zone 3 (P_H_S_H_)** | 6.56 | 64.01 | 18.87 | 65.38 |
| **Zone 4 (P_L_S_M_)** | 8.98 | 72.50 | 21.79 | 68.00 |
| **Zone 5 (P_M_S_M_)** | 8.42 | 75.91 | 24.18 | 70.79 |
| **Zone 6 (P_H_S_M_)** | 5.86 | 63.76 | 21.26 | 66.02 |
| **Zone 7 (P_L_S_L_)** | 2.98 | 59.52 | 12.35 | 57.24 |
| **Zone 8 (P_M_S_L_)** | 8.17 | 72.36 | 23.22 | 68.46 |
| **Zone 9 (P_H_S_L_)** | 6.35 | 68.00 | 20.88 | 65.57 |
| **Broader Precipitation Zones** | **Mean AMDP** | | **Std. Dev. of AMDP** | |
|  | **Avg. % increase** | **% area** | **Avg. % increase** | **% area** |
| **High Precipitation** | 6.18 | 65.38 | 20.63 | 65.73 |
| **Mod. Precipitation** | 7.60 | 71.55 | 22.34 | 68.52 |
| **Low Precipitation** | 6.10 | 63.39 | 19.00 | 63.29 |
| **High seasonality** | 5.03 | 59.92 | 17.92 | 62.70 |
| **Mod. seasonality** | 7.76 | 71.04 | 22.56 | 68.46 |
| **Low seasonality** | 7.07 | 69.70 | 21.48 | 66.48 |

**Supplementary Table 3**: Zone-wise comparison of average and standard deviation of mean AMDP values between pre- and post-1978 period.

| **Zones** | **Average** | | **Standard Deviation** | |
| --- | --- | --- | --- | --- |
|  | **Pre-1978** | **Post-1978** | **Pre-1978** | **Post-1978** |
| **Zone 1 (P_L_S_H_)** | 15.19 | 15.35 | 8.77 | 8.83 |
| **Zone 2 (P_M_S_H_)** | 51.81 | 54.28 | 20.99 | 22.22 |
| **Zone 3 (P_H_S_H_)** | 106.09 | 111.82 | 41.99 | 46.37 |
| **Zone 4 (P_L_S_M_)** | 13.75 | 14.76 | 3.87 | 4.33 |
| **Zone 5 (P_M_S_M_)** | 30.62 | 32.78 | 12.25 | 13.63 |
| **Zone 6 (P_H_S_M_)** | 93.85 | 98.93 | 15.11 | 20.72 |
| **Zone 7 (P_L_S_L_)** | 12.03 | 13.22 | 2.06 | 2.6 |
| **Zone 8 (P_M_S_L_)** | 42.77 | 45.48 | 29.24 | 31.06 |
| **Zone 9 (P_H_S_L_)** | 62.03 | 65.79 | 31.34 | 33.08 |

**Supplementary Table 4:** The Global, continental scale results of increase in PMP and the percentage of area showing the increase.

| **Continents** | **PMP** | |
| --- | --- | --- |
|  | **Avg. % increase** | **% area** |
| **Global** | 25.04 | 76.44 |
| **Asia** | 27.48 | 78.75 |
| **Europe** | 36.99 | 88.47 |
| **N. America** | 26.47 | 79.24 |
| **S. America** | 28.33 | 77.76 |
| **Australia** | 14.23 | 68.27 |
| **Africa** | 12.82 | 63.66 |

**Supplementary Table 5**: The zone-wise increase in PMP and the percentage of area showing the increase.

| **Precipitation Zones** | **PMP** | |
| --- | --- | --- |
|  | **Avg. % increase** | **% area** |
| **Zone 1 (P_L_S_H_)** | 14.69 | 64.89 |
| **Zone 2 (P_M_S_H_)** | 21.91 | 74.28 |
| **Zone 3 (P_H_S_H_)** | 19.09 | 74.08 |
| **Zone 4 (P_L_S_M_)** | 26.08 | 80.16 |
| **Zone 5 (P_M_S_M_)** | 34.83 | 82.60 |
| **Zone 6 (P_H_S_M_)** | 23.83 | 77.07 |
| **Zone 7 (P_L_S_L_)** | -2.83 | 41.31 |
| **Zone 8 (P_M_S_L_)** | 32.27 | 85.50 |
| **Zone 9 (P_H_S_L_)** | 29.01 | 79.58 |
| **Broad Precipitation Zones** | **PMP** | |
|  | **Avg. % increase** | **% area** |
| **High Precipitation** | 24.77 | 77.39 |
| **Moderate Precipitation** | 31.13 | 82.04 |
| **Low Precipitation** | 17.71 | 68.92 |
| **High seasonality** | 17.60 | 69.38 |
| **Moderate seasonality** | 28.80 | 80.13 |
| **Low seasonality** | 28.25 | 79.76 |


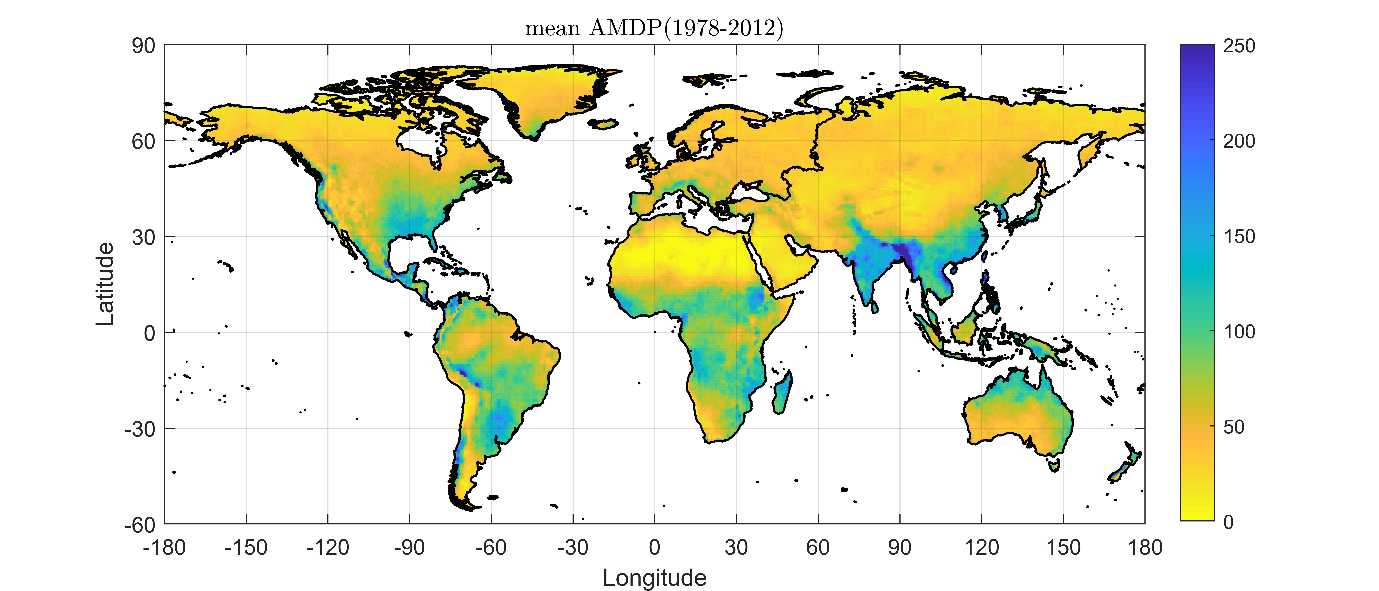

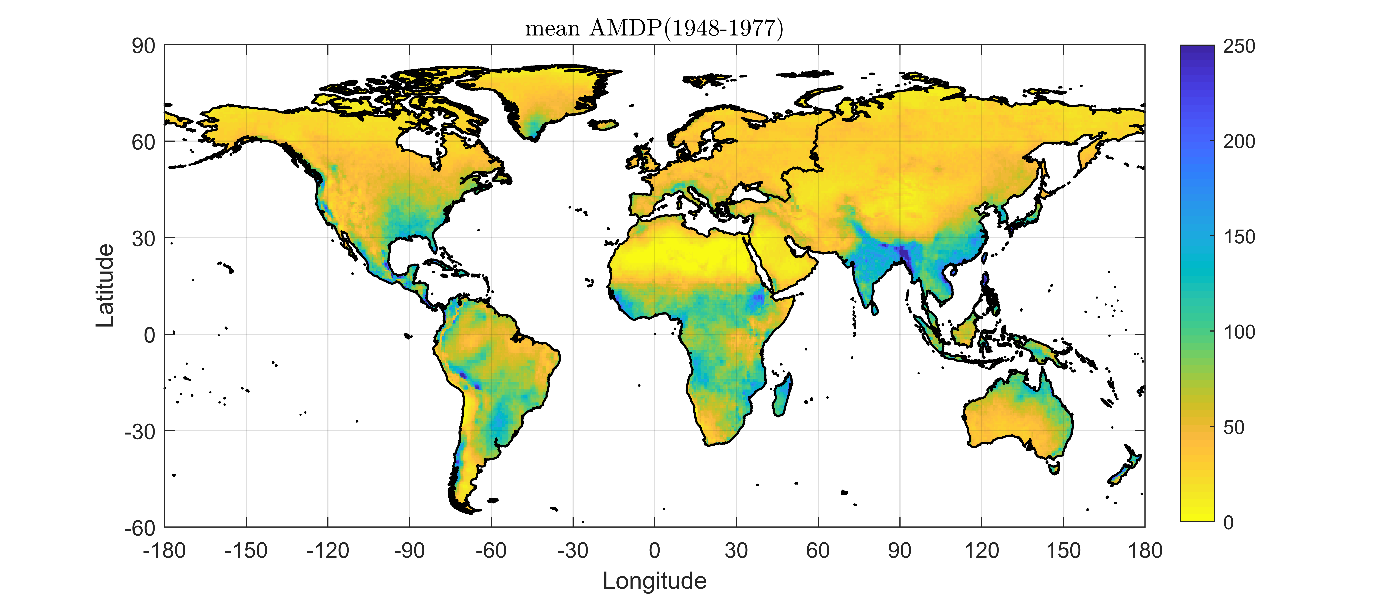


(a)

(b)

**Supplementary Figure 1:** Spatial Distribution of mean AMDP for (a) Pre-1978 period (1948-1977), and (b) post-1978 period (1978-2012). *The figure was produced using MATLAB software (version R2021a, URL:* [*https://in.mathworks.com*](https://in.mathworks.com)*).*


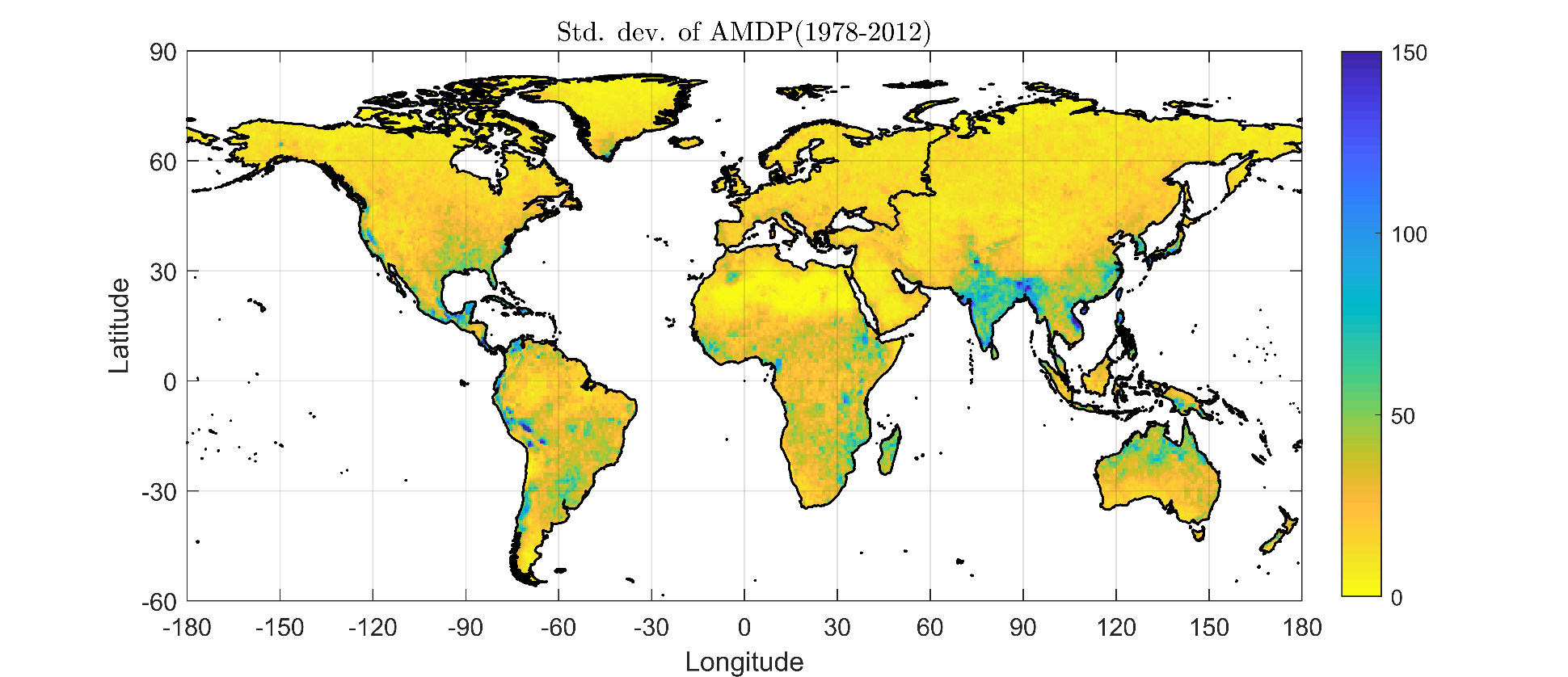

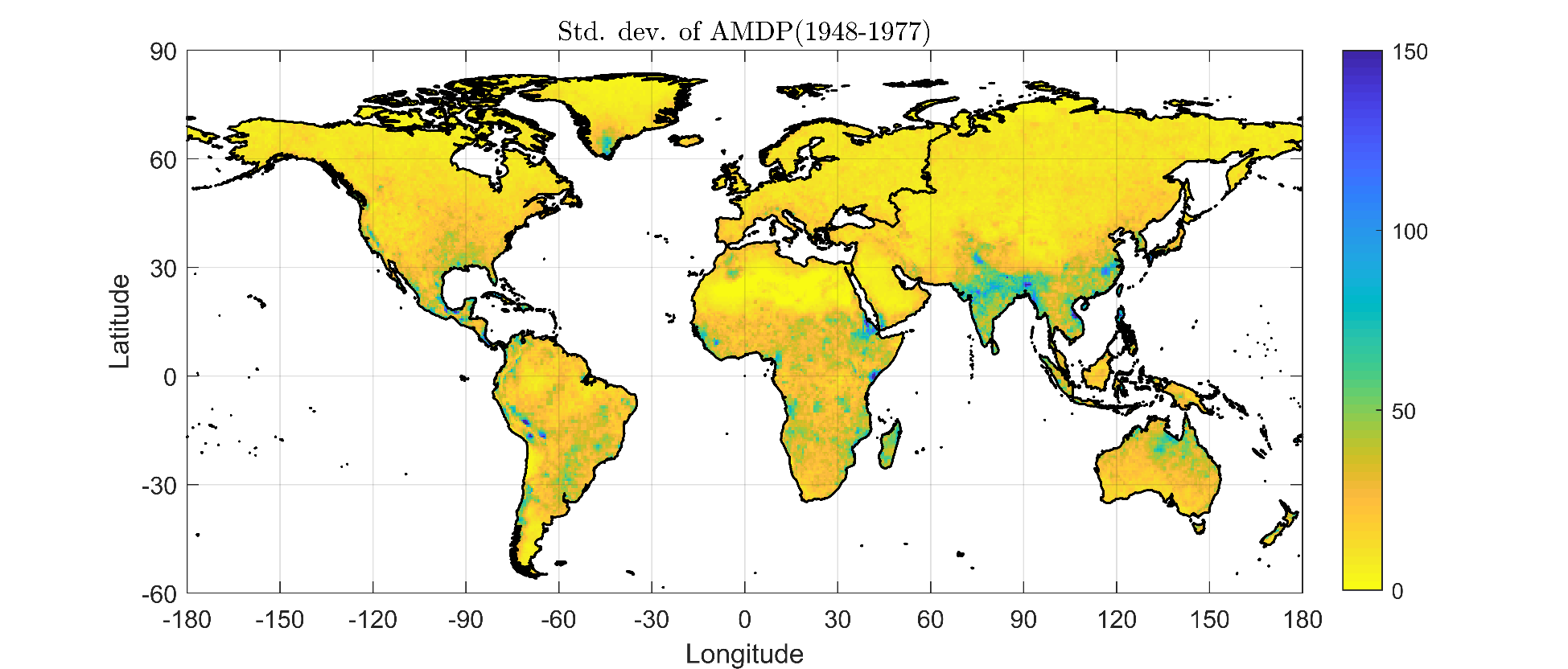


(a)

(b)

**Supplementary Figure 2:** Spatial Distribution of standard deviation of AMDP for (a) Pre-1978 period (1948-1977), and (b) post-1978 period (1978-2012). *The figure was produced using MATLAB software (version R2021a, URL:* [*https://in.mathworks.com*](https://in.mathworks.com)*).*


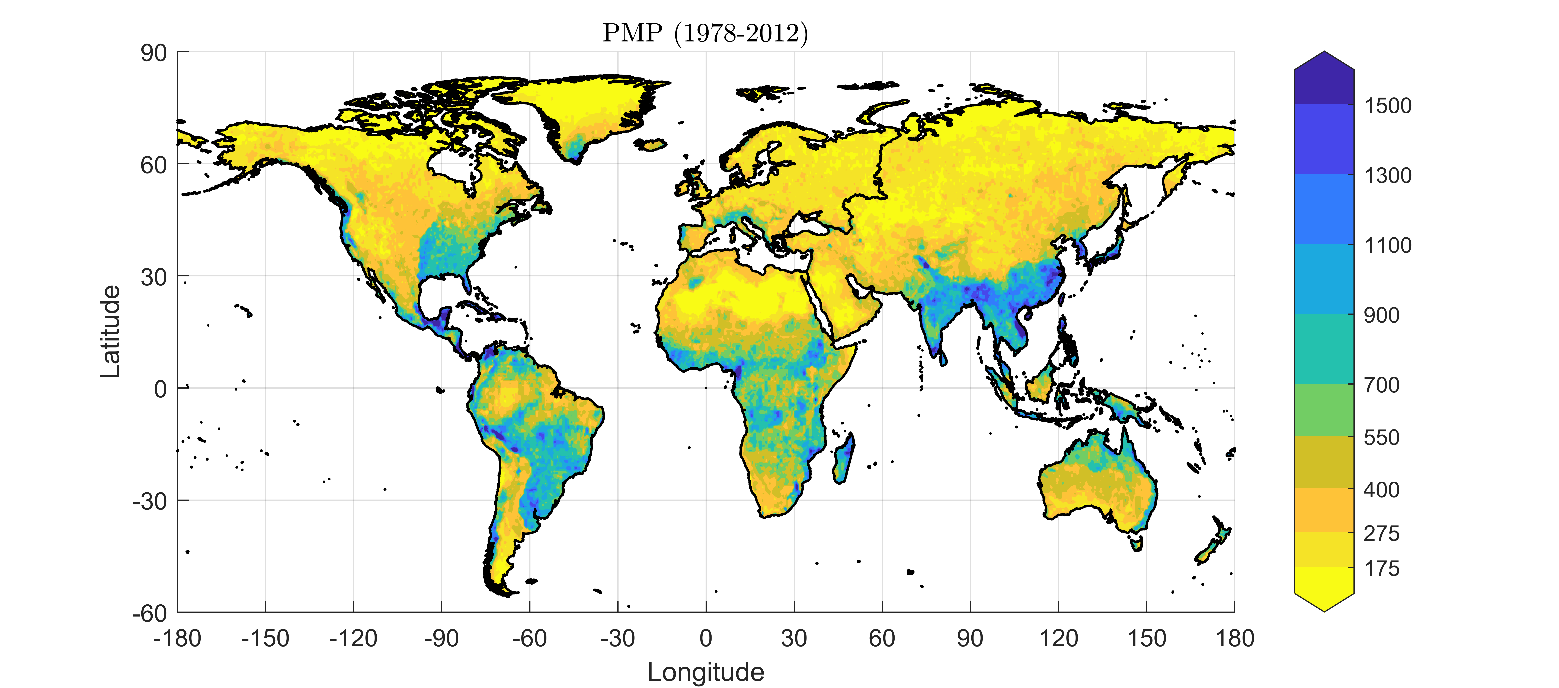

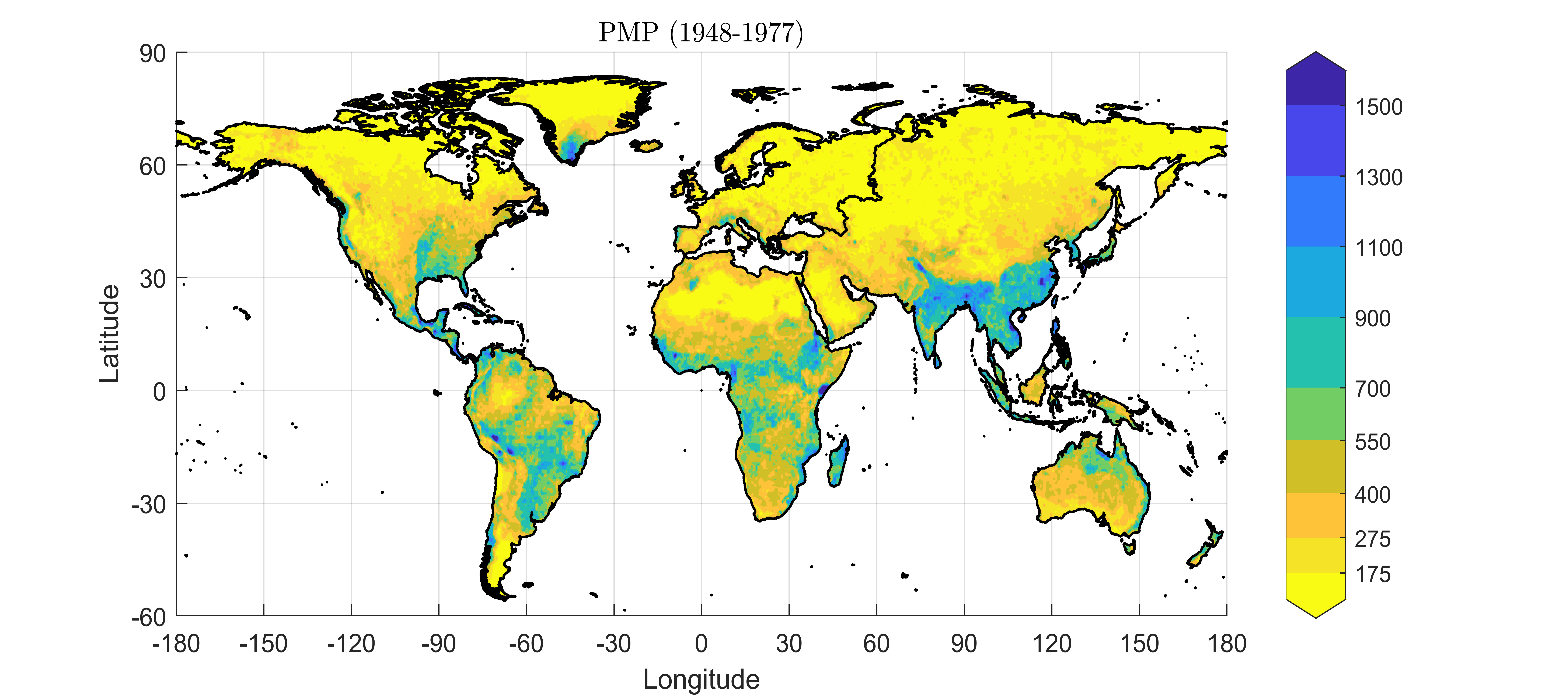


(a)

(b)

**Supplementary Figure 3:** Spatial Distribution of PMP for (a) Pre-1978 period (1948-1977), and (b) post-1978 period (1978-2012). *The figure was produced using MATLAB software (version R2021a, URL:* [*https://in.mathworks.com*](https://in.mathworks.com)*).*

**Supplementary Figure 4:** Global mean annual surface temperature and corresponding anomalies w.r.t. the base period 1951-1980. A clear increasing trend of temperature post-1978 (1978-2012) can be particularly noticed in contrast to pre-1978 period (1948-1977).

Single Envelope curve as per Hershfield method

Composite Envelope curve as per SM method

**Supplementary Figure 5:** A typical example of scatter plot and corresponding upper envelope curve for frequency factor; modified and reproduced from Sarkar and Maity, (2020).
